# Carboxymethyl Cellulose Surface Modification Alleviates the Toxicity of Fe-MOFs to Rice and Improves Iron Absorption

**DOI:** 10.3390/nano15050336

**Published:** 2025-02-21

**Authors:** Yuanbo Li, Yuying Tang, Yanru Ding, Yaping Lyu, Wenhao Su, Muhammad Nadeem, Peng Zhang, Yukui Rui

**Affiliations:** 1 Beijing Key Laboratory of Farmland Soil Pollution Prevention and Remediation, College of Resources and Environmental Sciences, China Agricultural University, Beijing 100193, China; 2 State Key Laboratory of Nutrient Use and Management, China Agricultural University, Beijing 100193, China; 3 Department of Environmental Science and Engineering, University of Science and Technology of China, Hefei 230026, China; lyp0213@ustc.edu.cn; 4 Department of Agricultural Engineering, College of Engineering, China Agricultural University, Beijing 100083, China; 5 China Agricultural University Professor Workstation of Tangshan Jinhai New Material Co., Ltd., Tangshan 063305, China; 6 China Agricultural University Professor Workstation of Wuqiang County, Hengshui 053000, China

**Keywords:** iron-based metal–organic frameworks, surface modification, rice, growth toxicity, carboxymethyl cellulose

## Abstract

Iron-based metal–organic frameworks (Fe-MOFs) are widely used for agricultural chemical delivery due to their high loading capacity, and they also have the potential to provide essential iron for plant growth. Therefore, they hold significant promise for agricultural applications. Evaluating the plant biotoxicity of Fe-MOFs is crucial for optimizing their use in agriculture. In this study, we used the natural biomacromolecule carboxymethyl cellulose (CMC) to encapsulate the Fe-MOF NH_2_-MIL-101 (Fe) (MIL). Through hydroponic experiments, we investigated the biotoxic effects of Fe-MOFs on rice before and after CMC modification. The results show that the accumulation of iron in rice is dependent on the dose and the exposure concentration of Fe-MOFs. CMC modification (MIL@CMC) can reduce the release rate of Fe ions from Fe-MOFs in aqueous solutions with different pH values (5 and 7). Furthermore, MIL@CMC treatment significantly increases the absorption of iron by both the aboveground and root parts of rice. MIL@CMC significantly alleviated the growth inhibition of rice seedlings and increased the aboveground biomass of rice under medium- to high-exposure conditions. Specifically, in rice roots, MIL induced a more intense oxidative stress response, with significant increases in the activities of related antioxidant enzymes (CAT, POD, and SOD) and MDA content. Our results demonstrated that the encapsulation of NH_2_-MIL-101(Fe) using CMC effectively alleviated oxidative damage and promoted the uptake and growth of iron in rice. These findings suggest that rational modification can have a positive effect on reducing the potential phytotoxicity of MOFs and improving their biosafety in agricultural applications.

## 1. Introduction

Metal–organic frameworks (MOFs) are a class of porous materials formed through coordination bonds between metal ions or metal clusters and organic ligands [1]. MOFs are characterized by their high specific surface area, adjustable pore structures, and unique physicochemical properties, which have garnered widespread attention in fields such as chemistry, physics, and materials science [2,3,4].

As an iron-based MOF, MIL-101(Fe)-NH_2_ has a unique structure and excellent physical and chemical properties, making it widely applicable in catalysis, adsorption, environmental remediation, and other fields [5,6,7,8]. Structurally, MIL-101(Fe)-NH_2_ features a large specific surface area and a hierarchical pore structure with both mesopores and supermesopores [9,10]. Although amination slightly decreases the pore size, the pore structure remains open, facilitating molecular transport. With the introduction of amino (-NH_2_) groups, the framework surface becomes more polar, enhancing interactions with hydrogen- or oxygen-containing molecules and improving adsorption capacity for specific compounds. MIL-101(Fe)-NH_2_ can adsorb various gases (such as CO_2_, H_2_, and CH_4_) and organic molecules [11,12]. The introduction of amino groups also increases the framework’s hydrophilicity. Additionally, as functional active sites, amino groups can enhance the material’s selectivity and catalytic efficiency for specific molecules [13,14]. The porous structure of MIL-101(Fe)-NH_2_ makes it suitable for drug molecule loading and controlled release. During drug release, the amino groups can modulate the release rate in a pH-responsive manner [15,16,17].

Iron is an essential trace element for plants, playing a crucial role in photosynthesis and enzyme reactions [18,19,20]. In traditional agriculture, iron deficiency can lead to chlorosis in plants, negatively affecting their growth. MIL-101(Fe)-NH_2_ can effectively release iron, helping to correct iron deficiency in crops [21,22,23]. Its microporous structure can improve the bioavailability of iron ions, ensuring that crops can adequately absorb iron. Iron-based metal–organic frameworks (MIL-101(Fe)-NH_2_) are increasingly showing potential in the agricultural field, especially as fertilizers. Due to the high specific surface area, adjustable pore structure, and biological importance of iron, MIL-101(Fe)-NH_2_ offers significant advantages in crop growth, nutrient release, and soil improvement [24,25]. However, the organic ligands in MIL-101(Fe)-NH_2_ can be released into the environment during degradation. These ligands, typically carboxylic acids or nitrogen-containing organic compounds, can pose environmental risks if they have complex structures or are resistant to biodegradation, potentially persisting in the environment for extended periods [24,26,27,28]. According to the Food and Agriculture Organization of the United Nations (FAO), global rice production has been rising over the past decade, with total production approaching 800 million tons in 2023. China, the world’s largest rice producer, reached approximately 207 million tons of rice in 2023 (https://www.fao.org/faostat/zh/#data/QCL/visualize (accessed on 11 Feburary 2025)). Despite this, the FAO report *The State of Food Security and Nutrition in the World 2023* still indicates that more than 864 million people worldwide face severe food insecurity. Therefore, crop safety must remain a top priority, particularly with regard to the use of agrochemicals.

Surface modification of MOFs can effectively slow their degradation. In this study, we used a biologically non-toxic natural material, CMC. CMC is an important cellulose derivative with good water solubility and thickening properties, and it is widely used in the food, pharmaceutical, daily chemical, textile, and paper industries [29,30,31]. Its excellent adhesion and film-forming abilities make it particularly advantageous for material coating applications [32]. On the one hand, CMC adheres well to various surfaces, providing a stable coating that prevents peeling and detachment. On the other hand, CMC’s strong film-forming capability enables the formation of a uniform and dense protective film on material surfaces [33,34]. This film can prevent direct contact with external factors, thereby extending the material’s shelf life and enhancing its durability [35]. This characteristic is especially suitable for coating irregularly shaped particles or objects, such as tablets, granular fertilizers, and seeds, ensuring an even and long-lasting coating [36,37,38]. This study aims to investigate, through hydroponic experiments, the effects of MOFs on rice seedling growth and development, comparing MIL with and without CMC coating. By comparing the biological impacts of CMC-coated and uncoated MOFs, we sought to evaluate CMC’s potential in mitigating the toxicity of MOFs and enhancing their agricultural applications.

## 2. Materials and Methods

### 2.1. Materials

FeCl_3_∙6H_2_O was purchased from Macklin Shanghai Macklin Biochemical Co., Ltd. (Shanghai, China). Fluorescein isothiocyanate (FITC), CMC (DS = 0.7), 2-Aminoterephthalic Acid (NH_2_-BDC), 1-Ethyl-3-(3-dimethylaminopropyl) carbodiimide HCl (EDC, 98%), and N-hydroxysuccinimide (NHS, 98%) were purchased from Shanghai Aladdin Biochemical Technology Co., Ltd. (Shanghai, China). SOD, CAT, POD, and MDA assay kits were purchased from Nanjing Jiancheng Bioengineering Co., Ltd. (Nanjing, China).

### 2.2. Experimental Design

The rice variety used was Shen Liangyou 5814, purchased from Hunan Ya Hua Seed Industry Co., Ltd., Hunan, China. Prior to germination, the rice seeds were disinfected by soaking in 0.5% NaClO (*v*/*v*) for 15 min, followed by three rinses with deionized water. The washed seeds were placed on trays lined with filter paper and incubated in the dark at a constant temperature of 26 °C for 4 days. After germination, the rice seeds were transferred to seedling trays containing deionized water and cultured for 3 days under conditions of 16 h of light and 8 h of darkness, with day and night temperatures of 28 °C and 25 °C, respectively [39]. Next, the seedlings were grown in half-strength nutrient solution for 6 days, with the solution being replaced every 3 days. Afterward, the seedlings were grown in full-strength nutrient solution until the two-leaf stage, at which point they were transferred to 250 mL white plastic bottles for cultivation. The composition of the full-strength nutrient solution was as follows: 1.43 mM (NH_4_)_2_SO_4_, 1.43 mM Ca(NO_3_)_2_·4H_2_O, 0.32 mM KH_2_PO_4_, 0.35 mM K_2_SO_4_, 1.65 mM MgSO_4_·7H_2_O, 0.1 mM Na_2_SiO_3_·9H_2_O, 9.1 μM MnCl_2_·4H_2_O, 0.074 μM (NH_4_)_6_Mo_7_O_24_·4H_2_O, 0.16 μM CuSO_4_·5H_2_O, 0.15 μM ZnSO_4_·7H_2_O, 18.5 μM H_3_BO_3_, and 35.8 μM EDTA-Fe.

Once the rice plants reached the three-leaf stage, Fe-MOF exposure treatments were initiated. The experiment included three treatments: MIL, MIL@CMC, and EDTA-Fe (Fe ion control). Each treatment group was divided into three concentrations: low, medium, and high. The concentrations for the MIL treatment group were set to 20, 100, and 250 mg/L. For the MIL@CMC and EDTA-Fe treatment groups, the molar amounts of Fe in each concentration gradient were equivalent to those in the MIL group. The control group was cultured using only the nutrient solution. The initial pH of all treatments was adjusted to 5.5 using 1 M HCl and 1 M NaOH, and the nutrient solution was replaced every 3 days.

### 2.3. Synthesis of MIL and MIL@CMC Experimental Design

Synthesis of MIL. MIL was synthesized by dissolving 2.757 g of NH_2_-BDC in 330 mL of DMF (N,N-dimethylformamide) solution. To this solution, 4.114 g of FeCl₃∙6H_2_O was added, and the mixture was sonicated for 30 min to ensure complete dissolution. The mixed solution was divided into four 100 mL PTFE-lined vessels, each filled with no more than 80 mL of the mixture. The PTFE liners were then placed in a reactor and heated at 120 °C for 20 h [40]. After cooling to room temperature, the precipitate was collected via centrifugation and washed three times with DMF and EtOH (ethanol), respectively. Finally, vacuum drying at 60 °C yielded MIL.

Synthesis of MIL@CMC. First, 200 mg of CMC was dissolved in 16 mL of MES buffer (pH 5.0, 20 mM). Then, 16 mL of a mixed aqueous solution of EDC and NHS (5 mM) was added. This was stirred at room temperature for 4 h [41]. Separately, 200 mg of MIL was dispersed in 80 mL of deionized water through sonication. The mixed CMC-NHS-EDC solution was added to the MIL solution, and stirring was continued for 24 h. The precipitate was collected via centrifugation, washed twice with deionized water, and vacuum-/freeze-dried to obtain MIL@CMC.

### 2.4. Determination of Rice Shoot Length, Root Length, and Biomass

Rice was harvested and washed briefly with deionized water (DI water). The plant height and root length of rice were determined using a millimeter ruler, while the biomass of fresh plants above and below ground was weighed using an analytical balance. A portion of fresh leaf tissue was taken for chlorophyll content determination. The rest of the plant samples were stored at −20 °C for subsequent analysis of antioxidant enzyme activities and nutrient contents.

### 2.5. Determination of Chlorophyll Content

At the time of harvest, the chlorophyll content in the plant leaves was measured according to the following method [39]. First, 40 mg of fresh rice leaves were taken and cut into julienne strips and then extracted by placing them in 95% (*v*/*v*) ethanol solution and immersing them in darkness, protected from light, for 24 h. Finally, the absorbance of the extracted solution was measured at 665 nm and 649 nm using a UV-vis spectrophotometer (TU-1900, China), and the chlorophyll a (Chla), chlorophyll b, and total chlorophyll content were obtained according to the following formula:

C_Chla_ = 13.95A_665_ − 6.88A_649_;

C_Chlb_ = 24.96A_649_ − 7.32A_665_; (1)

C_(Total Chlorophyll)_ = C_Chla_ + C_Chlb_ (2)

### 2.6. Determination of Antioxidant Enzyme Activity and MDA Content

Before determining the antioxidant enzyme activity or MDA content, plant tissue homogenates were first prepared. Briefly, 0.1 g of fresh rice leaf or root sample was placed in a 2 mL centrifuge tube, and grinding beads were added. After freezing with liquid nitrogen, the tissue was pulverized using a ball mill. Then, 1 mL of PBS buffer (pH 7.3–7.4, 0.01 mM) was quickly added to prepare a 10% tissue homogenate [42]. Based on the results of a pre-experiment, the 10% leaf tissue homogenate was diluted twice with PBS, resulting in a 5% tissue homogenate for the subsequent steps of the experiment. Tissue homogenates from the roots, which were needed for the next assay, were not diluted. The total protein concentration of the tissue homogenate was first determined, and then, using the appropriate kits, the antioxidant enzyme activity and MDA content were measured. Specifically, for the determination of CAT enzyme activity, the tissue homogenate was mixed with the reagents from the kit in a specific proportion and incubated at 37 °C for 1 min. The absorbance at 405 nm was then measured using a microplate reader (CF1524R, Thermo Fisher Scientific, Waltham, MA, USA). For the measurement of SOD and POD enzyme activities, the tissue homogenate was mixed with various reagents and incubated at 37 °C for 40 and 30 min, respectively, with absorbance readings taken at 550 nm and 420 nm, respectively. For MDA determination, the tissue homogenate mixture was heated at 95 °C for 40 min, and the absorbance was measured at 532 nm.

### 2.7. Determination of the Content of Mineral Elements

After completing the above biochemical experiments, the remaining rice samples were dried in an oven. First, they were treated at 105 °C for 30 min and then adjusted to 75 °C for drying until a constant weight was achieved. The dried plant samples were chopped, the aboveground samples (100 mg) or root samples (60 mg) were placed in an acid digestion tube, and then 6 mL of concentrated nitric acid (GR) was added. The samples were allowed to stand overnight at room temperature. Next, the samples were placed on a graphite heater (DigiBlock LabTech, Hangzhou Haoke Technology Co., Ltd., Hangzhou, China) for digestion. The sample was heated at 160 °C for 2 h. The solution was collected and diluted to ultrapure water to a volume of 50 mL and filtered through a 0.22 μm filter membrane [43]. The iron content was determined using inductively coupled plasma mass spectrometry (ICP-MS, Agilent, Santa Clara, CA, USA).

### 2.8. Distribution of Fe-MOFs in Plants

First, FITC-labeled MIL (FITC@MIL) was prepared. In brief, 200 mg of MIL was ultrasonically dispersed in 50 mL of ethanol. Then, 2 mL of FITC (1 mg/mL) ethanol solution was added. This was stirred at room temperature in the dark for 24 h. Then, it was centrifuged to collect the precipitate; the precipitate was washed three times with ethanol and freeze-dried to obtain FITC@MIL.

To confirm whether MIL can be absorbed by rice and to study its distribution after absorption, rice plants at the three-leaf stage were transferred to a nutrient solution containing 250 mg/L of FITC@MIL. The cultivation conditions were the same as those described in Section 2.2, and the exposure lasted for 6 days. A confocal laser scanning microscope (Olympus, FV3000, Tokyo, Japan) was used to observe the samples, with a laser excitation wavelength of 488 nm, and after 24 h, confocal laser scanning microscopy (CLSM) was used to observe the distribution of the samples in the roots, stems, and leaves of the rice.

### 2.9. Statistical Analysis

Two-way analysis of variance (ANOVA) and plotting were performed using the GraphPad 9.0 Prism software. Tukey’s method was used for multiple comparisons, and *p* < 0.05 was considered statistically significant.

## 3. Results and Discussion

### 3.1. Synthesis and Characterization of MIL and MIL@CMC

**Figure 1:**
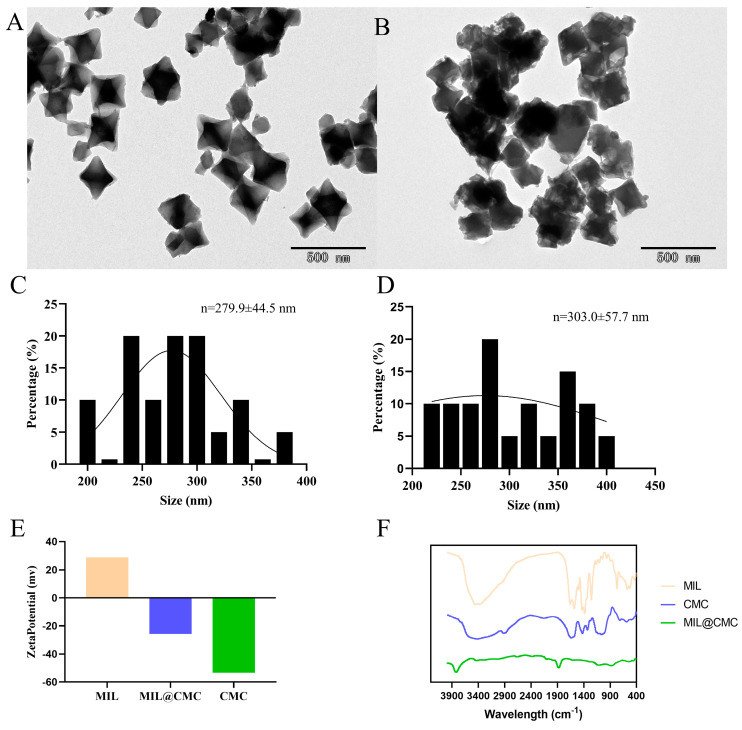
Characterization of the synthesized MOFs. TEM images of (**A**) the MIL and (**B**) the Fe- MIL@CMC. Particle size distribution images of (**C**) the MIL and (**D**) the MIL@CMC. Zeta-potential (**E**) and FTIR (**F**) characterizations of MOFs.

As shown in the TEM images, a clear morphological distinction is observed between MIL (Figure 1A and Figure S2(A1,A2)) and MIL@CMC (Figure 1B and Figure S2(B1,B2)). Figure S1 shows a schematic diagram of the preparation process for MIL and MIL@CMC. The particles of MIL exhibit a rhombic, regular crystal structure, suggesting a high degree of crystallinity. In the absence of additional modification, the particle size of MIL is relatively small, with individual particles measuring approximately 279.9 nm (Figure 1C). This regular morphology is a result of the formation of MIL [40]. In contrast, the particle size of MIL modified with CMC increased to approximately 303.0 nm (Figure 1D). The MIL@CMC particles exhibit a more irregular shape, a rougher surface, and signs of agglomeration, indicating that the introduction of CMC altered the surface morphology of MIL, leading to the loss of the regular particle arrangement [22,41]. Figure S2C,D show the hydration kinetic diameters of MIL and MIL@CMC, as measured with DLS, which are 859.2 nm and 840.27 nm, respectively. The discrepancy between the DLS data and TEM analysis, in which the particle size of MIL@CMC appears smaller in DLS measurements, can be attributed to several factors. Previous studies have shown that particle size measurements obtained through DLS tend to be larger than those derived from electron microscopy. We hypothesize that this discrepancy, along with the inherent heterogeneity of the materials, contributes to the observed differences in particle size between DLS and TEM analyses. The zeta potential data further suggest that CMC modification significantly alters the surface charge of MIL. The zeta potential of unmodified MIL is +28.8 mV, while that of the modified material is −25.7 mV (Figure 1E). A larger absolute zeta potential generally correlates with increased stability and a reduced tendency for agglomeration. Therefore, as shown in Figure 1B, more pronounced agglomeration is observed in the modified material.

Figure 1F presents the infrared absorption spectra of two MOF materials (MIL and MIL@CMC) obtained through FTIR analysis of different samples. The FTIR spectrum of MIL exhibits characteristic absorption peaks associated with the MOF structure. Peaks in the range of 1000–1600 cm^−1^ are likely attributed to the stretching vibrations of C=C and C=O in the organic ligand, while peaks in the lower wavenumber region (<1000 cm^−1^) may be associated with the vibrations of metal–ligand coordination bonds. The spectrum of CMC exhibits typical absorption peaks of cellulose, with a broad peak in the region of 3200–3500 cm^−1^, which is likely associated with the O-H stretching vibration, a characteristic feature of the hydroxyl groups in carboxymethyl cellulose. The peak around 1600 cm^−1^ may correspond to the C=O stretching vibration in the carboxymethyl group [44]. MIL@CMC, a composite of MIL and CMC, combines the characteristic peaks of both MIL and CMC in its spectral curve. However, the peak positions and intensities in MIL@CMC differ slightly from those in individual MIL and CMC, indicating an interaction between MIL and CMC. The spectrum of MIL@CMC exhibits peaks that are characteristic of both MIL and CMC; however, subtle shifts in peak positions and intensities, particularly for the O-H and C=O bands, suggest interactions between MIL and CMC. This interaction suggests the formation of a stable composite structure through hydrogen bonding or coordination bonding, which could influence the physico-chemical properties of the composite material, making it suitable for specific applications such as drug delivery or catalysis [45,46].

### 3.2. Release of Fe Ions from Fe-MOFs in a Water Solution

**Figure 2:**
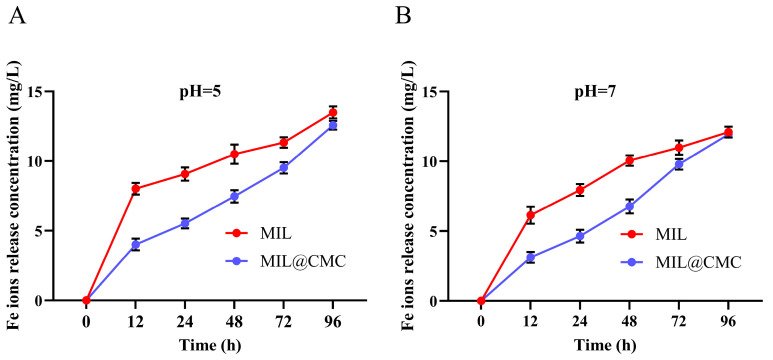
Cumulative release of Fe ions from MOFs in a water solution at pH 5 (**A**) and pH 7 (**B**). Data are expressed as the mean ± SD (n = 3).

As shown in Figure 2A, in an aqueous solution with a pH of 5.0, the Fe ion release from MIL reached 7.68 ppm at 12 h, 9.24 ppm at 24 h, 10.50 ppm at 48 h, 11.34 ppm at 72 h, and 13.62 ppm at 96 h. The release values of MIL@CMC at the same time points are 6.20 ppm (12 h), 7.93 ppm (24 h), 9.95 ppm (48 h), 10.85 ppm (72 h), and 11.83 ppm (96 h), respectively. The Fe ion release from MIL increases over time, and at each time interval, the release from MIL is higher than that from MIL@CMC, indicating that the CMC-modified material has a certain inhibitory effect on the release of Fe ions at lower pH levels.

At pH 7, the release of Fe ions from MIL increases with time, although the overall release is significantly lower than that at pH 5.0. Similarly to the results at pH 5, the release from MIL@CMC is lower than that from MIL at each time point (Figure 2B). Across all time points, the rate of Fe ion release from MIL was generally higher than that from MIL@CMC, indicating that CMC modification reduced the release rate of Fe ions. The release of Fe ions increased gradually over time, showing a positive correlation between time and the amount released.

Although CMC modification reduced the release of Fe ions at both pH values, the inhibitory effect was relatively small at pH 7, which may indicate that CMC has better stability and structure in non-acidic conditions. Acidic conditions could promote the release of iron ions. Marcuello et al. found that increasing humidity improves the adhesion between lignocellulose and cellulose nanocrystals prepared using glucomannan. In addition, an increase in humidity decreases the reduced Young’s modulus of lignocellulosic films while increasing the water adsorption capacity of this polymer film [47]. Nikiforov et al. prepared dialdehyde cellulose through chemical modification of short flax fiber using sodium metaperiodate. It was shown that the adsorption capacity of the modified prepared cellulose for Cu^2+^, Cd^2+^, and Fe^2+^ was increased by about 2.4–2.9 times [48]. Therefore, in addition to CMC potentially slowing the release of Fe ions by enhancing the stability of MOFs, another possible mechanism is that carboxymethyl cellulose may have some adsorption effect on Fe ions. As a result, fewer Fe ions are detected from the release of MIL@CMC compared to MIL.

### 3.3. Effects of MIL and MIL@CMC on Rice Growth and Photosynthesis

**Figure 3:**
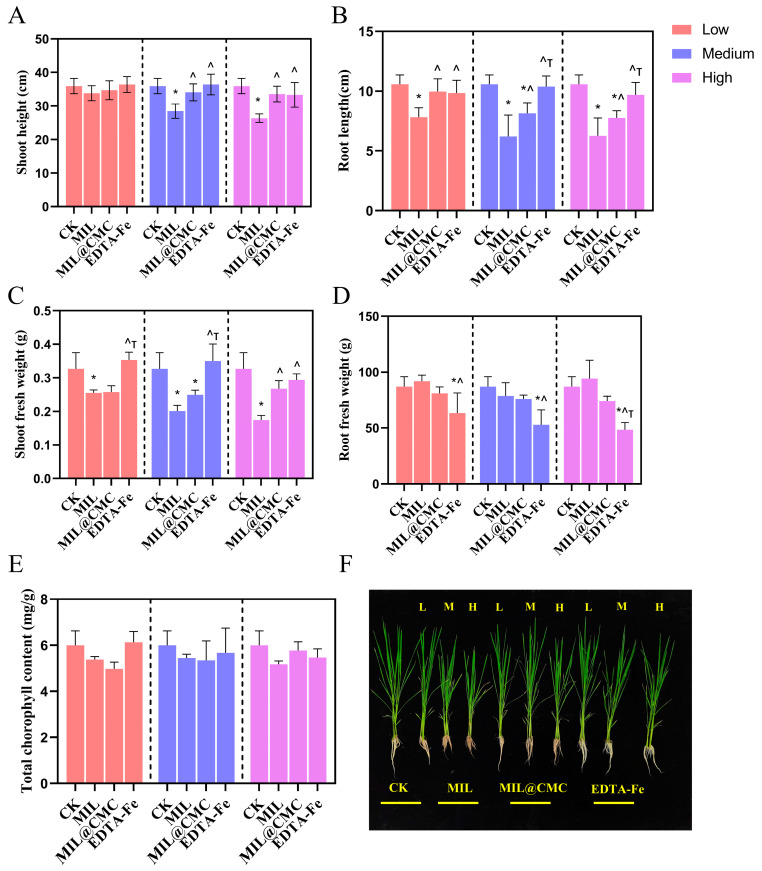
The plant height (**A**), root length (**B**), fresh weight of the aboveground parts (**C**), fresh weight of the roots (**D**), and chlorophyll content (**E**) of rice seedlings after 15 days of exposure to varying concentrations of MIL or MIL@CMC. Rice seedlings exposed to different concentrations of MIL or MIL@CMC for 15 days (**F**). Data are expressed as the mean ± SD (n = 3). (* *p* < 0.05 vs. CK, ^ *p* < 0.05 vs. MIL, ^Τ^ *p* < 0.05 vs. MIL@CMC.)

Figure 3A,B illustrate the effects of different treatment conditions on the plant height and root length of rice seedlings at low, medium, and high levels, respectively. In general, plant height increased significantly with increasing treatment levels. Specifically, in the MIL, MIL@CMC, and EDTA-Fe groups, plant height increased significantly with increasing treatment levels. There were significant differences between treatment groups, especially at high treatment levels, where the plant heights of the MIL@CMC and EDTA-Fe groups were significantly greater than those of the control group. Similarly, root length increased significantly with increasing treatment levels. This trend was particularly notable in the MIL@CMC and EDTA-Fe groups, where increasing treatment levels were closely associated with a significant increase in root length. Figure 3B also reveals significant differences between treatment groups, particularly at high treatment levels. All treatments (especially the EDTA and EDTA-Fe treatments) showed a notable increase in root length compared to the control group (CK). The MIL@CMC and EDTA-Fe groups outperformed other treatments in terms of both shoot and root length, particularly at high treatment levels, indicating a substantial growth-promoting effect on the plants. EDTA, as a chelating agent, may promote plant growth by improving the availability of iron and facilitating the uptake of other essential nutrients [49,50,51]. Most of the differences between treatment groups were statistically significant, particularly at high treatment levels, further demonstrating that MIL@CMC was more effective in promoting rice plant height and root length than both the control and MIL treatment groups. The fresh weight of the above-ground parts of rice decreased at all treatment concentrations, especially at medium and high levels, with a significant decrease in the MIL treatment group (Figure 3C). In the MIL@CMC treatment group, the fresh weight of the aboveground parts did not show significant changes with increasing concentration. It is worth noting that at the highest concentration, the fresh weight of the aboveground parts was significantly higher than that in the MIL treatment group, although it was still lower than that in the control group. Both types of Fe-MOFs generally inhibited the fresh weight of aboveground parts. For root fresh weight, there was no significant change in root biomass in the MIL and MIL@CMC groups compared with the CK group (Figure 3D). However, in the EDTA-Fe treatment group, the root biomass at all concentrations was significantly lower than that in the CK and MIL groups. At the highest concentration exposure, the root biomass in the EDTA-Fe group was lower than that in all other treatment groups.

The total chlorophyll content showed minimal variation across the different treatment groups, exhibiting a relatively stable trend. In contrast to the biomass of aboveground parts and roots, changes in treatment levels had a minor impact on the total chlorophyll content (Figure 3E). This indicates that the treatments had limited influence on the chlorophyll content, suggesting either that they did not significantly alter the plants’ photosynthetic capacity or that the plants had reached a saturation point in this regard, rendering chlorophyll content largely unaffected by the treatment variations.

In addition to measuring the Fe content in rice tissues, we also measured the contents of Mg, Mn, and Zn (Figure S3). These three metal elements are nutrients included in the rice nutrient solution. The results indicate that, compared to the control group, MIL significantly reduced the content of Mn and Mg in both the aboveground parts and roots of rice (Figure S3A,C). Only under high-concentration MIL@CMC exposure conditions was the Mn content in rice leaves significantly inhibited. In contrast to the trend observed for Mn content, exposure to any concentration of MIL@CMC significantly reduced the Mg content in the aboveground parts of rice compared with the control group (Figure S3B,D). Moreover, only high-concentration Fe-MOF exposure inhibited Zn absorption by rice seedlings (Figure S3E,F). Overall, compared to MIL, MIL@CMC enhanced the absorption of Mg and Mn by rice seedlings. This is consistent with the changes in Fe content in rice tissues. We speculate that this is due to the selective adsorption of Mg and Mn by CMC, which leads to their increased absorption by rice along with Fe.

### 3.4. Uptake of Fe by Rice Aboveground and Root Systems

**Figure 4:**
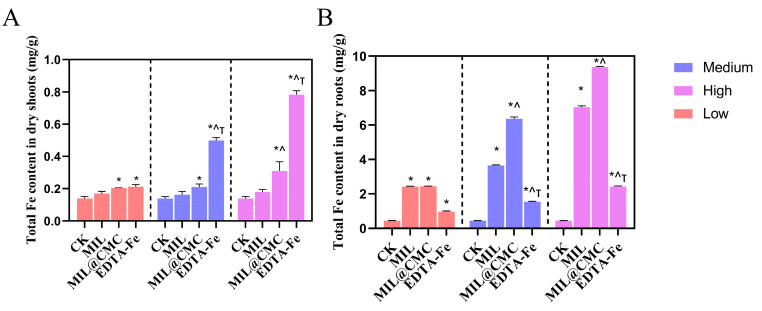
Fe content in the aboveground parts (**A**) and roots (**B**) of rice seedlings after 15 days of exposure to different concentrations of MIL or MIL@CMC. Data are expressed as the mean ± SD (n = 3). (* *p* < 0.05 vs. CK, ^ *p* < 0.05 vs. MIL, ^Τ^ *p* < 0.05 vs. MIL@CMC.)

Figure 4A displays the Fe content in the aboveground parts of rice in treatments with different concentrations of Fe-MOFs or EDTA. The Fe content of the MIL treatment group was 0.169 mg/g, which was slightly higher than that of the control group. It increased to 0.203 mg/g at medium concentration and further increased to 0.213 mg/g at high concentration. This indicates that MIL treatment has a modest effect on increasing Fe content in the aboveground parts of rice. As the concentration increases, the Fe content gradually increases, but the change is not substantial. In the MIL@CMC treatment group, the Fe content was 0.162 mg/g at low concentration, which was slightly lower than that of MIL. It increased to 0.211 mg/g at medium concentration and significantly rose to 0.500 mg/g at high concentration. This suggests that MIL@CMC has a notable effect at high concentrations, with the enhanced Fe absorption potentially being due to CMC modification, which may improve the Fe uptake. Compared with the MOF treatment groups, the Fe content in the EDTA treatment group increased the most, with an Fe content of 0.179 mg/g at low concentration, which was slightly higher than that of MIL@CMC, and it reached 0.310 mg/g and 0.784 mg/g at medium and high concentrations, respectively. This indicates that EDTA has a strong promoting effect on Fe absorption by rice, especially at high concentrations. Overall, from low to high concentration, the Fe content gradually increased, with the EDTA and MIL@CMC treatments showing the greatest increments. EDTA had the most substantial effect, suggesting that its role as a chelating agent significantly improved Fe absorption by rice. MIL@CMC showed a secondary effect, possibly due to enhanced plant absorption resulting from CMC-modified MIL at high concentrations.

As shown in Figure 4B, the Fe content in the MIL treatment group was significantly higher than that in the control group, with a clear increase in the total Fe content as the exposure concentration increased, indicating a noticeable promoting effect of MIL treatment on Fe uptake in rice roots. Compared to the MIL treatment, the MIL@CMC treatment further increased the total Fe content in plants, especially at the medium and high concentration levels, where the Fe content increased significantly. This suggests that the combined use of MIL and CMC has a favorable effect on Fe uptake. In the EDTA-Fe treatment group, the Fe content in rice roots at medium and high concentration levels also increased significantly compared with the control, though it was notably lower than that in the MIL and MIL@CMC treatment groups. This suggests that while EDTA-Fe effectively chelates Fe, its effect on enhancing Fe uptake by rice roots is less pronounced than that of the MIL and MIL@CMC treatment groups.

### 3.5. Effects of Fe-MOF Exposure on the Antioxidant System in Rice

**Figure 5:**
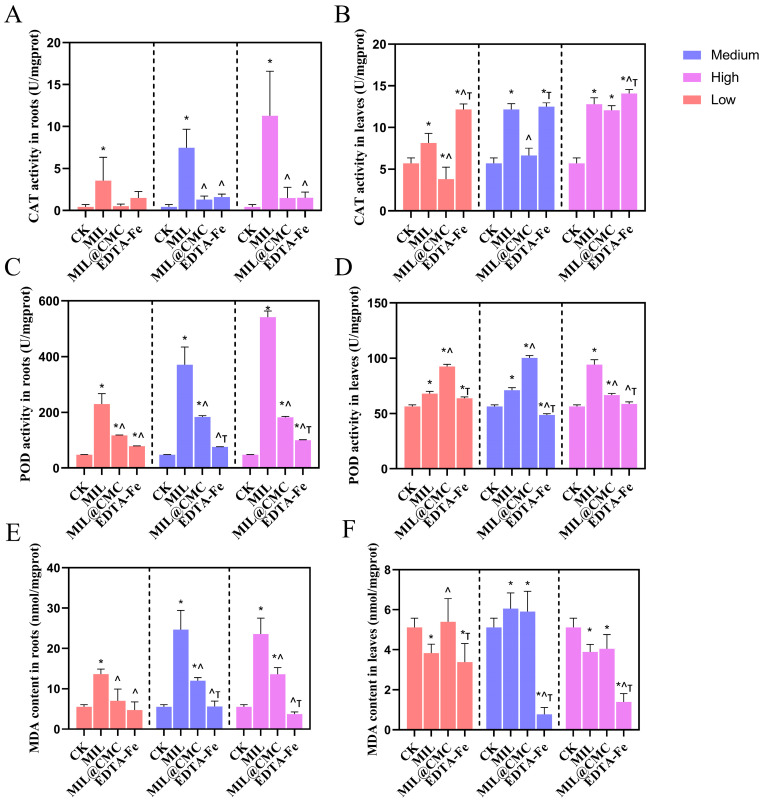
CAT enzyme (**A**,**B**) and POD enzyme (**C**,**D**) activities and MDA contents (**E**,**F**) in rice leaves and roots after 15 days of exposure to different concentrations of MIL or MIL@CMC. Data are expressed as the mean ± SD. Data are expressed as the mean ± SD (n = 3). (* *p* < 0.05 vs. CK, ^ *p* < 0.05 vs. MIL, ^Τ^ *p* < 0.05 vs. MIL@CMC.)

The CAT enzyme is an antioxidant enzyme primarily responsible for decomposing hydrogen peroxide (H_2_O_2_) within cells and reducing oxidative stress damage to plants [52,53]. An increase in CAT activity typically indicates that a plant is experiencing severe oxidative stress and is enhancing its CAT activity to alleviate this stress [54,55]. In each exposure group, the CAT activity in the roots was lower in the EDTA-Fe and MIL@CMC groups across various concentrations. For the EDTA-Fe group, the CAT activity did not change much, with concentration increases having minimal impact, remaining below 2 U/mgprot. At medium and high concentrations, the CAT activity in the roots of the MIL@CMC-treated rice increased noticeably compared to low-concentration exposure. In the MIL treatment group, the CAT activity in rice roots at all concentrations was significantly higher than that in the control group and other treatment groups (Figure 5A). The CAT enzyme activity in the leaves of the control group was significantly lower than that in the treatment groups. In both the MIL and EDTA-Fe groups, the CAT activity in the roots increased significantly with increasing exposure concentrations, reaching nearly 15 U/mgprot at high concentration levels (Figure 5B). This suggests that oxidative stress in the roots increased notably with higher treatment levels, necessitating higher CAT activity to mitigate oxidative stress. In the MIL@CMC group, the CAT activity exceeded that of the control only at high concentration levels. Overall, the CAT activity in the MIL treatment group increased significantly with increasing concentration in both roots and leaves, indicating a strong impact on the plant’s antioxidant enzyme system, which may have been due to increased oxidative stress, prompting the plant to respond by increasing its CAT activity. The CAT activity in both roots and leaves followed a similar trend, increasing with increasing treatment levels, but the CAT levels in leaves were generally higher than in roots. This may be due to leaves being the primary site of photosynthesis, where they are exposed to greater environmental oxidative stress and, thus, require a stronger antioxidant defense system to protect the cells [56].

In the low-concentration treatment, the POD enzyme activity in rice roots of each treatment group was low but remained significantly higher than that in the CK group (Figure 5C,D). At medium and high concentrations, the POD enzyme activity of each treatment group significantly increased, particularly in the MIL treatment group. At the highest treatment level, the POD activity was approximately 600 U/mgprot. This suggests that this treatment had the greatest impact on root oxidative stress. In contrast, changes in leaf POD enzyme activity were less pronounced with the increase in exposure concentration. At low and medium concentrations, the POD activity in the leaves of the MIL@CMC treatment group was significantly higher than that in other groups. However, with a high exposure concentration, the POD enzyme activity in rice leaves was highest in the MIL treatment group.

MDA is a product of lipid peroxidation in cell membranes and is commonly used to indicate the extent of oxidative damage to plant cell membranes [57,58]. In rice roots, at low treatment levels, the MDA content in each treatment group varied slightly, remaining at a low level (around 5–10 nmol/mgprot), indicating minimal lipid oxidation damage to the root membranes under these conditions (Figure 5E,F). At medium and high treatment levels, particularly in the MIL treatment group, the MDA content increased significantly, with the highest value approaching 20 nmol/mgprot. This suggests that oxidative stress in the roots intensified with increasing treatment concentration, leading to greater membrane damage. Notably, in the MIL@CMC treatment group, the MDA content in rice roots was significantly lower than that in the MIL treatment group. In rice leaves, the MDA content was lower than that in the roots, consistently around 5–10 nmol/mgprot in each treatment group. With medium and high exposure concentrations, there was no significant difference in MDA content between the MIL@CMC and MIL treatment groups in rice leaves. Only with the low exposure concentration did the MDA content in the MIL@CMC group significantly exceed that in the MIL group.

### 3.6. Distribution of Fe-MOFs in Rice Roots, Stems, and Leaves

**Figure 6:**
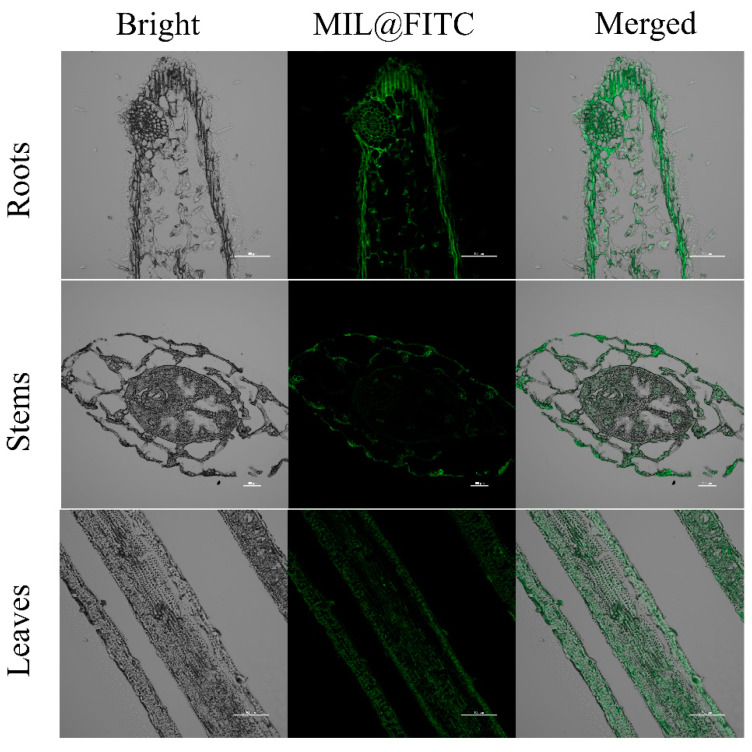
CLSM images of different parts of rice seedlings (stems, roots, and leaves) treated with MIL@FITC.

Figure 6 displays confocal microscopy images of rice roots, stems, and leaves, where green fluorescence (FITC) is overlaid on a gray background, indicating the distribution of FITC-labeled MIL materials within different tissue structures. In the root images, fluorescence is primarily concentrated in the peripheral and central areas of the root, likely corresponding to the root structure and differentiation regions. The fluorescent signals may highlight the root epidermis or vascular tissue, suggesting a concentrated distribution of target molecules within these tissues. The stem images reveal a relatively complex structure, with fluorescence concentrated in the vascular bundle area, particularly in the central vascular system of the stem. The leaf images exhibit a distinct linear pattern, with fluorescent signals aligned along the leaf veins, indicating regions with high fluorescence markings within the vascular system of the leaf.

MOFs have shown potential for transport within plants, following pathways often used by nutrients, nanoparticles, or small molecules [59,60]. Based on existing research, the transport mechanisms of MOFs in plants generally involve several key pathways. (1) Root Uptake and Translocation: MOFs can enter plants primarily through root absorption from the soil or a hydroponic solution [59,60]. Once absorbed, they move through the apoplastic or symplastic pathways in the roots. In the apoplastic pathway, MOFs travel around the cells, through cell walls, and through intercellular spaces until they reach the endodermis. Here, the Casparian strip acts as a selective barrier, channeling them into the symplastic pathway if they are able to cross the cell membrane [61]. Within cells, MOFs can utilize plasmodesmata (tiny channels connecting plant cells) to move symplastically from cell to cell. (2) Xylem Transport: Once MOFs reach the root vascular tissues, they can enter the xylem, which conducts water and solutes upward from roots to stems and leaves [23]. Transpiration, the process by which water evaporates from the leaf surface, creates negative pressure in the xylem that pulls water (and dissolved or suspended materials such as MOFs) upward through the plant. This allows MOFs to reach the aerial parts of the plant, including the stems and leaves. (3) Phloem Redistribution: In addition to xylem transport, MOFs may be loaded into the phloem tissue, which redistributes nutrients and other substances throughout the plant. This transport mechanism is driven by a pressure flow created by osmotic differences, enabling the MOFs to move from sources (such as leaves) to sinks (areas of growth or storage, such as roots or fruits). While xylem transport is unidirectional (upward), phloem movement allows for more versatile, multidirectional distribution, potentially enhancing the availability of MOFs in various parts of the plant [62,63]. (4) Endocytosis and Cellular Localization: Some studies have shown that nanoparticles can be internalized by plant cells through endocytosis, allowing them to enter cellular compartments [64,65,66]. Within the cells, MOFs may localize to specific organelles, such as chloroplasts or vacuoles, potentially facilitating targeted nutrient delivery or enhancing photosynthesis. The internalization and specific cellular localization could vary based on the size, surface charge, and chemical composition of the MOFs. Overall, these mechanisms highlight the potential of MOFs for efficient transport and delivery within plant systems, providing insights into their role in enhancing nutrient uptake and improving plant health.

## 4. Conclusions

To summarize, we effectively reduced the phytotoxicity of MOFs by encapsulating NH_2_-MIL-101(Fe) with the natural biomacromolecule CMC. The results demonstrated that Fe-MOFs are absorbed by the root system and are also translocated to the aboveground parts. Compared to exposure to MIL, MIL@CMC enhanced the uptake of elemental Fe by both the rice root system and leaves, significantly increasing the aboveground biomass of rice. The oxidative stress response to Fe-MOFs was more pronounced in the root system than in the leaves. The activities of SOD, CAT, and POD enzymes were significantly higher in the root system of MIL-treated rice seedlings compared to MIL@CMC-treated seedlings, indicating that MIL caused more pronounced oxidative damage to the rice root system. We believe that the mechanism by which Fe-MOF toxicity is mitigated through modification involves two main aspects. One mechanism is the reduction of the oxidative damage suffered by rice, while the other is the enhancement of its biomass by improving nutrient uptake. Since Fe-MOFs are highly diverse, and the effects of different types of Fe-MOFs on phytotoxicity may vary, more systematic studies are needed to explore their toxicity differences in the future. Additionally, considering the various ways in which MOFs can be utilized as agrochemicals (e.g., foliar spraying or root exposure), it is essential to study their uptake, transformation, and fate processes from the environment to the plant in order to analyze the biological effects of these different application methods on plants. The results of these studies will be valuable in further optimizing the use of MOFs and ensuring their biosafety.

## Data Availability

Data are contained within the article.

## Supplementary Materials

The following supporting information can be downloaded at: https://www.mdpi.com/article/10.3390/nano15050336/s1, Figure S1: Illustration for the Synthesis of MIL@CMC. Figure S2. TEM images of (A) the MIL and (B) the Fe- MIL@CMC at 100 and 200 nm scale. Hydration kinetic diameters distribution images of (C) the MIL and (D) the MIL@CMC. Figure S3. Mn (A,B), Mg (C,D), Zn (E,F) content in the above-ground parts and roots of rice seedlings after 15 days of exposure to different concentrations of MIL or MIL@CMC. Data was expressed as mean ± SD (n = 3). (* *p* < 0. 05 vs. CK, ^ *p* < 0. 05 vs. MIL, Τ *p* < 0. 05 vs. MIL@CMC).
