# Supplementary material for: Carboxymethyl Cellulose Surface Modification Alleviates the Toxicity of Fe-MOFs to Rice and Improves Iron Absorption
Source: Nanomaterials (Basel). 2025 Feb 21;15(5):336. doi: 10.3390/nano15050336 (PMC11901664; doi:10.3390/nano15050336)
Supplement: Supplementary file 1 [file nanomaterials-15-00336-s001.zip › nanomaterials-3461022-supplementary.pdf]

Supporting Information

for

Carboxymethyl Cellulose Surface Modification Alleviates the Toxicity of Fe-MOFs to Rice and Improves Iron Absorption

Yuanbo Li [1,2,†], Yuying Tang [1,2,†], Yanru Ding [1,2], Yaping Lyu [3], Wenhao Su [4], Muhammad Nadeem [1,2], Peng Zhang [3,*] and Yukui Rui [1,2,5,6,*]

[1] Beijing Key Laboratory of Farmland Soil Pollution Prevention and Remediation, College of Resources and Environmental Sciences, China Agricultural University, Beijing 100193, China
[2] State Key Laboratory of Nutrient Use and Management, China Agricultural University, Beijing 100193, China
[3] Department of Environmental Science and Engineering, University of Science and Technology of China, Hefei 230026, China; lyp0213@ustc.edu.cn
[4] Department of Agricultural Engineering, College of Engineering, China Agricultural University, Beijing 100083, China
[5] China Agricultural University Professor Workstation of Tangshan Jinhai New Material Co., Ltd., Tangshan 063305, China
[6] China Agricultural University Professor Workstation of Wuqiang County, Hengshui 053000, China
* Correspondence: zhangpeng1987@ustc.edu.cn (P.Z.); ryk@cau.edu.cn (Y.R.)
† These authors contributed equally to this work.

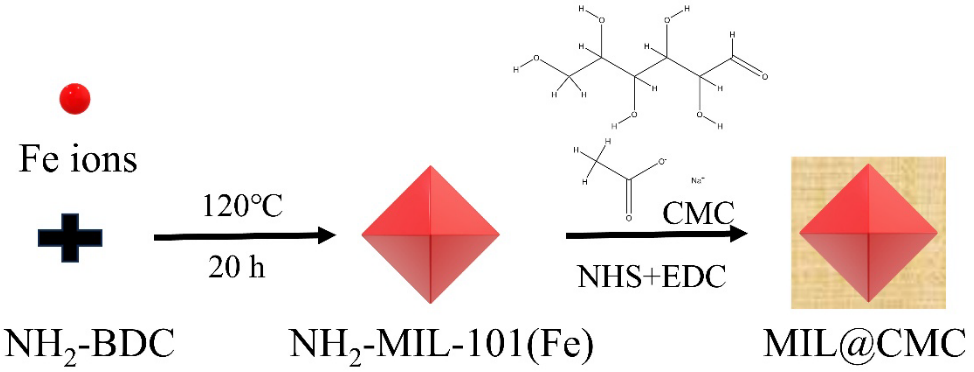

Figure S1. Illustration for the Synthesis of MIL@CMC.

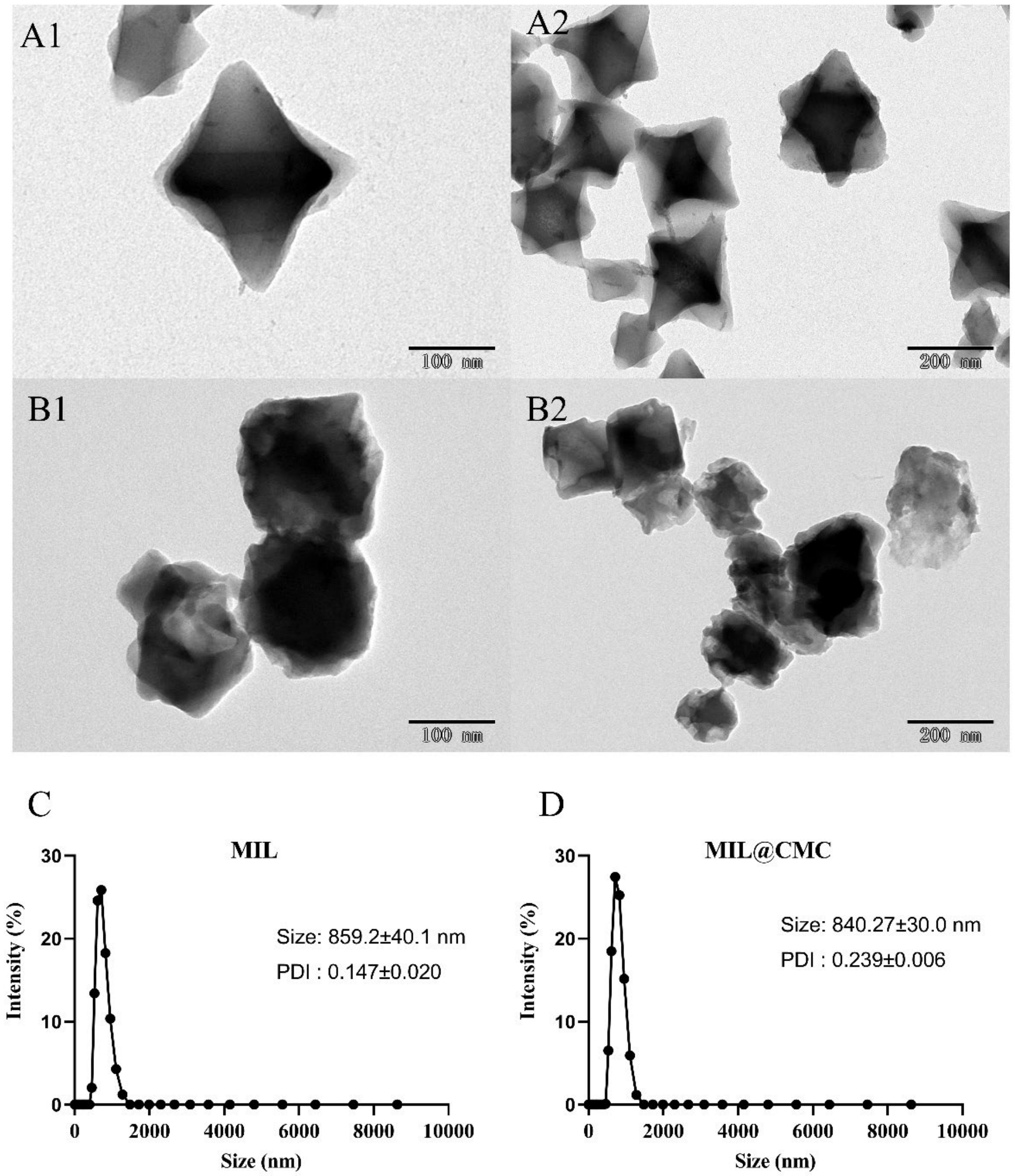

Figure S2. TEM images of (A) the MIL and (B) the Fe- MIL@CMC at 100 and 200 nm scale. Hydration kinetic diameters distribution images of (C) the MIL and (D) the

MIL@CMC.

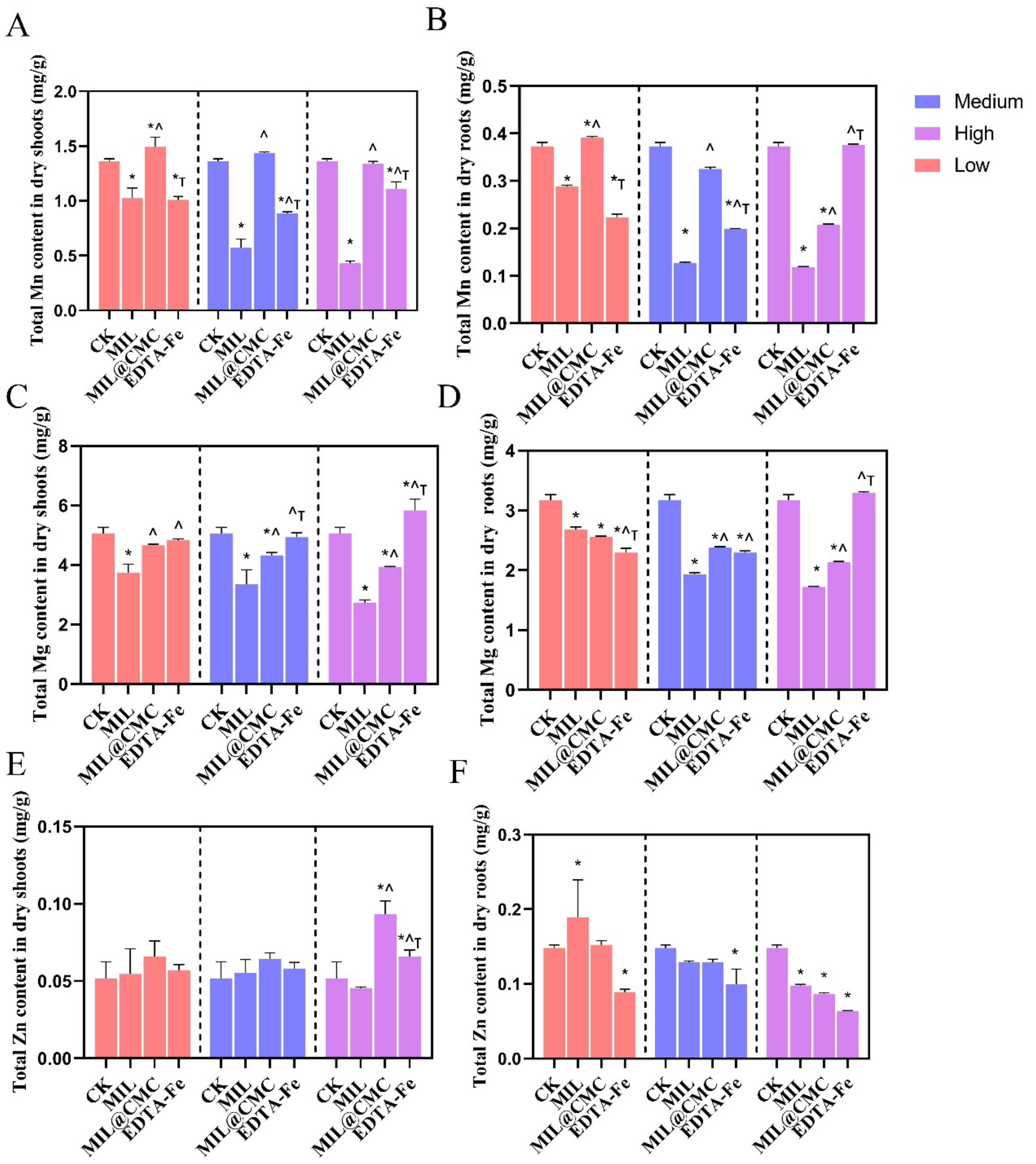

Figure S3. Mn (A and B), Mg (C and D), Zn (E and F) content in the above-ground parts and roots of rice seedlings after 15 days of exposure to different concentrations of MIL or MIL@CMC. Data was expressed as maen ± SD (n=3). (*p < 0. 05 vs. CK, ^p < 0. 05 vs. MIL, Tp < 0. 05 vs. MIL@CMC).
